# TaCAMTA4, a Calmodulin-Interacting Protein, Involved in Defense Response of Wheat to *Puccinia triticina*

**DOI:** 10.1038/s41598-018-36385-1

**Published:** 2019-01-24

**Authors:** Yuelin Wang, Fengju Wei, Hui Zhou, Na Liu, Xiaonan Niu, Chao Yan, Lifeng Zhang, Shengfang Han, Chunyan Hou, Dongmei Wang

**Affiliations:** Key Laboratory of Hebei Province for Plant Physiology and Molecular Pathology, College of Life Sciences, Hebei Agriculture University, Baoding, 071001 China

## Abstract

Leaf rust caused by *Puccinia triticina* is one of the main diseases affecting wheat (*Triticum aestivum*) production worldwide. Calmodulin (CaM) was found involved in the early stage of signal transduction pathway in response to *P*. *triticina* in wheat. To study the function and molecular mechanism of calmodulin (CaM) in signal transduction of wheat against *P*. *triticina*, we cloned a putative calmodulin-binding transcription activator (*TaCAMTA4*), and characterized its molecular structure and functions by using the CaM-encoding gene (*TaCaM4-1)* as a bait to screen the cDNA library from *P*. *triticina* infected wheat leaves. The open reading frame of *TaCAMTA4* was 2505 bp encoding a protein of 834 aa, which contained all the four conserved domains of family (CG-1 domain, TIG domain, ANK repeats and CaM-binding domain). TaCaM4-1 bound to TaCAMTA4 by the C-terminal CaM-binding domain in Ca^2+^-dependent manner in the electrophoretic mobility shift assay (EMSA). Bimolecular fluorescence complementation (BiFC) analysis indicated that the interaction of TaCAMTA4 and TaCaM4-1 took place in the cytoplasm and nucleus of epidermal leaf cells in *N*. *benthamiana*. The expression level of *TaCAMTA4* genes was down-regulated in incompatible combination after *P*. *triticina* infection. Furthermore, virus-induced gene silencing (VIGS)-based knockdown of *TaCAMTA*4 and disease assays verified that silencing of *TaCAMTA*4 resulted in enhanced resistance to *P*. *triticina* race 165. These results suggested that *TaCAMTA*4 function as negative regulator of defense response against *P*. *triticina*.

## Introduction

Plants have developed sophisticated mechanisms to sense and respond to various stresses so as to adapt to their environment. Ca^2+^ signaling pathway plays an important role in plant defense responses to biotic stresses (e.g., pathogens, hormones, etc.) and abiotic stresses (e.g., cold, hot, drought, etc.)^1^. Intracellular Ca^2+^ transient increase is an early signal to induce defense responses in plants^2–4^. Specific Ca^2+^ signals could be recognized by Ca^2+^ receptors and then transferred to other proteins involved in Ca^2+^ signaling pathways^5^. Calmodulin (CaM), the primary cellular Ca^2+^ receptor, recognizes and binds specific Ca^2+^ signals through its EF-hand domain, and transfers Ca^2+^ signals by interacting with the calmodulin-binding domains (CaMBD) of target calmodulin-binding proteins (CaMBPs). Many CaMBPs have been identified to play important roles in defense responses to stress in plants^4,6–8^.

Calmodulin-binding transcription activator (CAMTA), one of the CaMBPs, also known as signaling-responsive protein (SR), is a highly conserved transcription factor family^4,6–8^ in plants and other multicellular organisms (e.g., mammals, insects, etc.)^1^. The CAMTA family has four highly conserved domains: the CG-1 DNA-binding domain which specifically recognizes CGCG box and CGTG box in promoter regions of target genes^1^ and contains a typical bipartite nuclear locating signal (NLS) necessary for nuclear importation; the transcription factor immunoglobin (TIG) domain which is a putative nonspecific DNA-binding domain, a protein-protein interacting domain containing ankyrin (ANK) repeats, and a CaM-binding domain which consists of IQ domain and CaMB domain^9^.

CAMTAs function in growth and development, as well as stress defenses, in plants^10^. In *A*. *thaliana* genome, six CAMTA genes (*AtCAMTA1-6* or *AtSR1-6*) were identified, and their expressions could be induced by stresses including cold and heat, abscisic acid (ABA), ethylene, methyl jasmonate (MJ), H_2_O_2_ or salicylic acid (SA)^11^, respectively. It has been shown that AtCAMTA1 closely relates with auxin^12^ and drought signaling^13^ and AtCAMTA3 plays a role in cold tolerance^11^. CAMTAs regulate resistance-related genes expression in biotic stress defense processes^9,14,15^, the DNA-binding domain of AtCAMTA3 could bind the CGCG box in the promoter of *EDS1* gene to suppress *EDS1* expression, and Ca^2+^/CaM binding was necessary for AtCAMTA3 to negatively regulate *EDS1* expression and plant immunity^16^.

We have long engaged in the study of the signal transduction mechanism of wheat defense response against *P*. *triticina*. Pharmacological tests using Ca^2+^ signal blockers and activators^17–19^, subcellular localization of cytoplasmic Ca^2+^ ^20^, and cytoplasmic Ca^2+^ dynamic detection of elicitor stimulated wheat mesophyll protoplasts^21^ showed that Ca^2+^ play an important role in the signal transduction pathway in wheat resistance against *P*. *triticina*. Real-time quantitative RT-PCR (qRT-PCR) and western blotting showed that, in the early stage of incompatible interactions between wheat and *P*. *triticina*, the expression of *TaCaM4* was the highest among the eleven *CaMs*. Furthermore, *CAM4*-silenced plants showed relatively weak HR (hypersensitive response) signals compared with control. Suppression subtractive hybridization (SSH) indicated that the expression of *TaCaM4* increased at 8 hours after *P*. *triticina* infection^22^. All the previous results demonstrated that TaCaMs were involved in the early stage of incompatible interaction processes, and play an important role in the wheat resistance signal transduction pathway against *P*. *triticina*. However, the putative molecular mechanism underlying the involvement of CAMs in wheat- *P*. *triticina* interaction signal pathway still remain unclear.

In this study, we cloned a putative CAMTA gene by screening cDNA library from wheat leaves infected *P*. *triticina* and designated as *TaCAMTA4* for its highest homology with *AtCAMTA4*, and analyzed their gene structures. Furthermore, EMSA was employed to determine the CaM binding motif. We also further verified the interaction by Bimolecular fluorescence complementation (BiFC) analysis in tobacco epidermal leaf cells. The expression profiles of *TaCAMTA4* gene were analyzed using quantitative real-time RT-PCR (qRT-PCR). Finally, using VIGS (Virus-induced gene silencing) -based knockdown, we revealed that *TaCAMTA4* may negatively regulated wheat basic resistance to *P*. *triticina* race 165.

## Results

### Screening of TaCaM4-1 interacting proteins and amplification of *TaCAMTA4* full length cDNA by RACE

**Figure 1:**
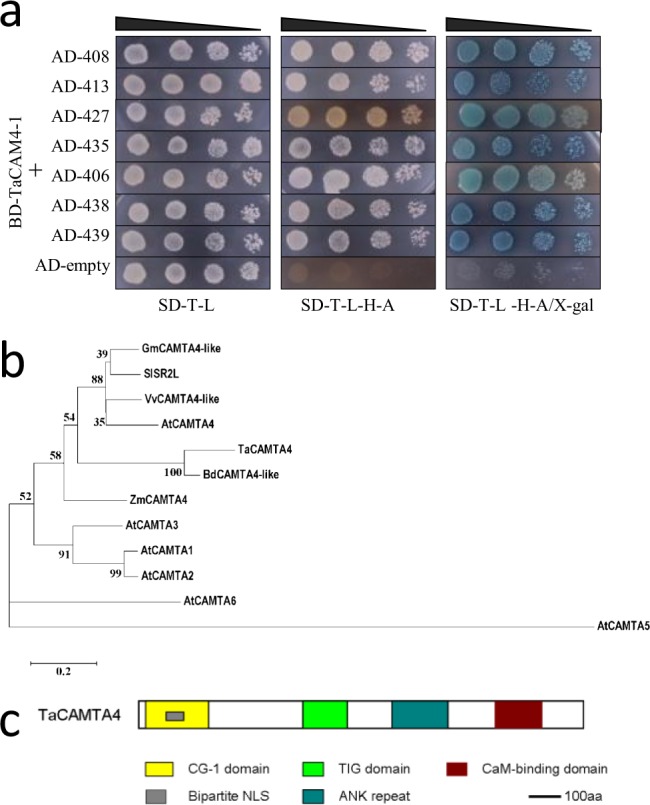
Screening of TaCaM4-1 interacting proteins (**a**) Interaction tests using yeast two-hybrid assays between TaCAM4-1 and prey proteins. Yeasts harboring TaCAM4-1 and prey proteins were placed in different liquid concentrations on control medium SD/-Trp/-Leu and selection medium SD/-Trp/-Leu/-His/-Ade. For negative controls, pGADT7 without insert TaCAM4-1 was used (pGBKT7-TaCAM4-1 + pGADT7). Experiments were performed three times and a representative result is shown. The full-length blots are presented in Supplementary Fig 1. (**b**) Phylogenetic analyses of TaCAMTA4 and its homologs from different plant species. The TaCAMTA4 protein sequence was used to perform BLAST searches against the National Center for Biotechnology Information database. TaCAMTA4 and its homologs identified in different organisms were aligned. Gm, *Glycine max*; Vv, *Vitis vinifera*; At, *Arabidopsis thaliana*; Sl, *Solanum lycopersicum*; Zm, *Zea mays* L; Bd, *Brachypodium distachyon*; Ta, *Triticum aestivum*. (**c**) TaCAMTA4 conserved domains prediction. CG-1, specific CGCG box-containing DNA sequences; TIG, transcription factor ImmunoGlobin; CaM-binding, calmodulin-binding domain; ANK repeat, ankyrin repeats; Bipartite NLS, nuclear locating signal; aa, amino acids.

To explore the roles of wheat *TaCaM4-1* in *P*. *triticina* infection, pGBKT7-*TaCaM4-1* bait vector was used for screening of wheat yeast two-hybrid cDNA library, resulting in 45 positive clones. To verify that the proteins interact with TaCAM4-1, prey plasmids in the positive clones were extracted and transformed into yeast AH109 together with bait plasmid pGBKT7-*TaCaM4-1* in one-on-one method for interaction detection to eliminate false positive clones. Yeast cells with both plasmids (cell number 406, 408, 413, 427, 435, 438 and 439) grew on the selection medium, while the cells with either plasmid was absent, indicating that these proteins interact with TaCAM4-1 in yeast cells (Fig. 1a). Among the 7 candidate genes encoding CaM-binding protein obtained by yeast two-hybrid, an 896-bp sequence (termed Code. 408) was highly homologous to CAMTA genes, with the typical transcription factor characteristics. In order to get the full length cDNA of Code. 408, RACE was conducted to amplify the 5′ end of the cDNA fragment. The resulting PCR amplicon of the full length cDNA was 2704 bp and the open reading frame was 2505 bp (S1 Figure).

Blast analysis on NCBI showed that the Code.408 gene was homologous to CAMTA genes in *Glycine max*, *Vitis vinifera*, *Solanum lycopersicum*, *Zea mays* and *Brachypodium distachyon*. Phylogenetic analysis of our gene and six CAMTAs in *A*. *thaliana* (*AtCAMTA1-6* or *AtSR1-6*) showed that our gene was highly homologous to *AtCAMTA4* (Fig. 1b). As a result, this gene was named *TaCAMTA4* and deposited in GenBank (accession No. KC686696). Prediction of conserved domains in TaCAMTA4 indicated that TaCAMTA4 contained all the conserved domains of the CAMTA family: CG-1 domain, TIG domain, ANK repeats, and CaM-binding domain (Fig. 1c). The CG-1 domain is a 130-amino acid highly conserved domain and contains a bipartite NLS necessary for nuclear import. CG-1 domain can bind CGCG box in the promoter region of genes. TIG domain widely exists in endocellular transcription factors and cell surface receptors and functions in interaction with DNAs or proteins. ANK repeats exist in the form of ankyrin tandem repeat but the number of tandems is various in different genes and different species. ANK repeats may function in protein-protein interaction. The probable CaM-binding domain of TaCAMTA4 was found at the C- terminal which could function in CaM recognizing and binding. The bioinformatic analysis suggested that TaCAMTA4 could be a novel member of the wheat CAMTA family.

### Interactions between TaCAMTA4 and TaCAM4-1

Previous studies have showed CaM-binding domains of CaMBPs were mostly located on the C-terminal^23^. Bioinformation analysis revealed that the probably CaM-binding domain of TaCAMTA4 was also on the C-terminal. In order to detect the binding between CaM-binding sites of TaCAMTA4 and TaCaM4-1, two peptide sequences in TaCAMTA4, as AVQAAGRIQATFRVFSLKKKKQKALQNRGS (666–695 aa) and

**Figure 2:**
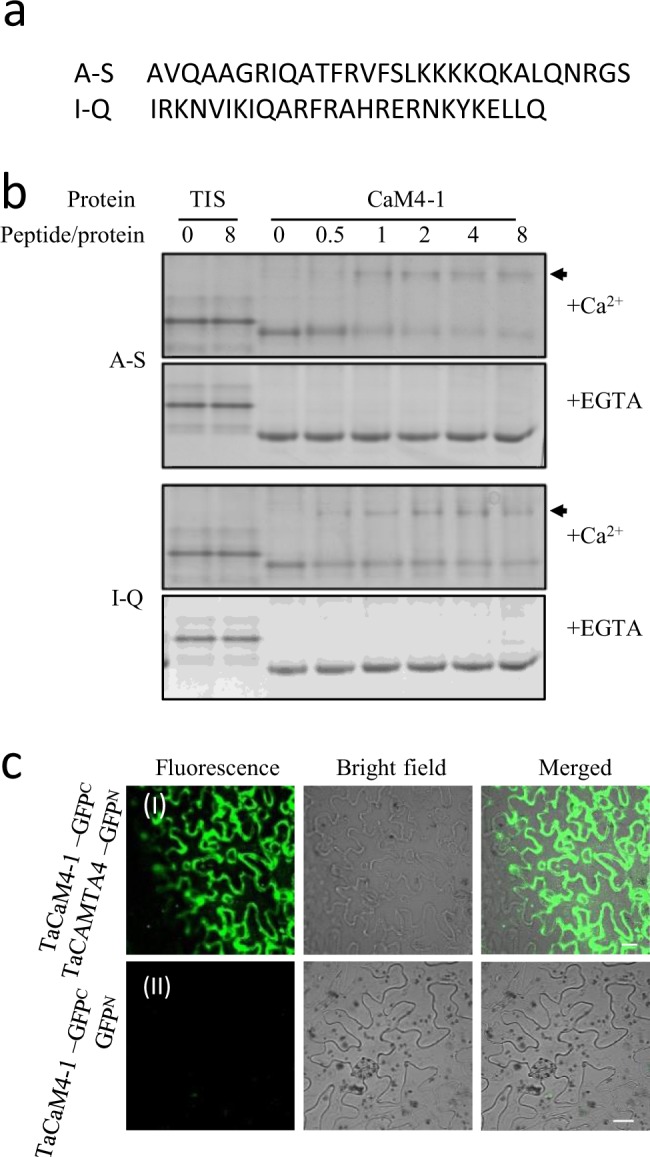
TaCAMTA4 interacted with TaCaM4-1 in EMSA and BiFC assays. (**a**) Two motif sequences of TaCAMTA4. Two synthetic peptides termed A-S and I-Q. (**b**) Two motifs of TaCAMTA4 interacted with CaM *in vitro* with EMSA. A-S peptide and I-Q peptide were mixed with prokaryotic expressed TaCaM4-1 on peptide/protein ratio of 0, 0.5, 1, 2, 4 and 8, respectively, in present of 1 mM Ca^2+^ or 2 mM EGTA, and detected by native PAGE after reaction 1 hour at room temperature. TIS (trypsin inhibitor) was used as negative control. Arrows indicate the bands of peptide/CaM complex. The full-length gels are presented in Supplementary Figure 2. (**c**) TaCAMTA4 interacted with TaCAM4-1 by BiFC assays in *N*. *benthamiana*. (I). The coding regions of *TaCAMTA4* and *TaCaM4-1* genes were fused with the N-terminal (GFP^N^) and C-terminal (GFP^C^) region of GFP, respectively. The recombinant constructs pSPYCE(MR)- *TaCaM4-1*/pSPYNE(R)173- *TaCAMTA4* were co-transformed into *N*. *benthamiana*. (II). The combination of *TaCaM4-1* -GFP^C^ and GFP^N^ was used as a negative control. The results were observed 48 h after infiltration, Scale bar, 20 μm.

IRKNVIKIQARFRAHRERNKYKELLQ (725–750 aa) were chosen to conduct the CaM-binding analysis *in vitro* (Fig. 2a). The two synthetic peptides termed A-S and I-Q were respectively mixed with prokaryotic expressed TaCaM4-1 at peptide/CaM molar ratios of 0, 0.5, 1, 2, 4 and 8 in reaction buffer and spontaneously reacted at room temperature for 1 hour and then were detected by native PAGE. The results showed that, in the presence of 1 mM Ca^2+^ and in the absence of peptides, TaCaM4-1 appeared as a single band. After A-S or I-Q peptide was added, a second slower mobility band appeared indicating formation of the peptide/CaM complex. And the I-Q/CaM complex band appeared when peptide/CaM molar ratio reached 0.5, whereas A-S/CaM complex band did not appear until peptide/CaM molar ratio reached 1, suggesting the binding characteristics in different regions of the CaM-binding sites. In the presence of 2 mM EGTA, no complex band appeared for either A-S or I-Q (Fig. 2b). The results suggested that TaCAMTA4 could bind to TaCaM4-1 by the C-terminal CaM-binding domain in Ca^2+^-dependent manner.

The interaction between TaCAMTA4 and TaCaM4-1was further confirmed using a BiFC assay, where the N- and C-terminal GFP fragments were fused to TaCAMTA4 and TaCaM4-1, respectively, and co-expressed in *N*. *benthamiana* leaves. The fluorescent signals of GFP indicated that interaction between TaCAMTA4 and TaCaM4 co-located in cytoplasm and nucleus (Fig. 2c).

### *TaCAMTA4* was decreased after *P. triticina* infection

**Figure 3:**
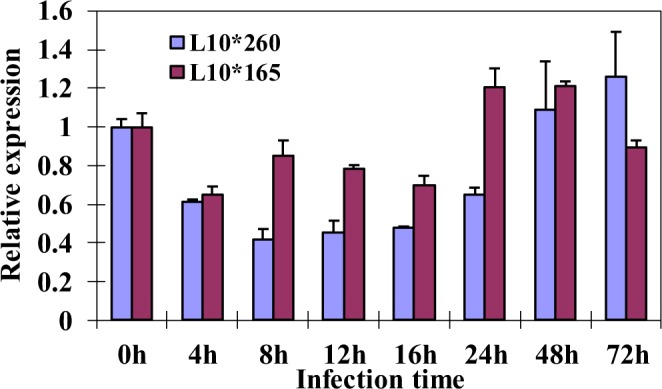
Expression of *TaCAMTA4* in different combination plants after *P*. *triticina* infection. The transcription levels of wheat *TaCAMTA4* were detected by qRT-PCR in both incompatible and compatible combinations at different time points after *P*. *triticina* infection. Mock treatment combination was used as control. Constitutively expressed gene *EF-1-α* was used as a reference. Results are means ± SD with three replicates.

The involvement of CaM in *P*. *triticina* infection has been well confirmed. In order to clarify whether CaM-binding TaCAMTA4 regulates the interaction process, the transcription levels of *TaCAMTA4* were detected by qRT-PCR in both incompatible and compatible combinations at different time after *P*. *triticina* infection. In the incompatible combination, the expression levels of *TaCAMTA4* decreased after *P*. *triticina* infection and showed the lowest level at 8 hours after infection, about 40% of the expression level at 0 h. The transcription level rised at 24 h and got back to 0 h level at 48 hours after infection. However, in the compatible combination, the transcription of *TaCAMTA4* slightly decreased and the change was not obvious as that in the incompatible combination (Fig. 3). The results indicated that down regulation of *TaCAMTA4* expression is required for resistance of wheat against *P*. *triticina*.

### TaCAMTA4 negatively regulate the defense response of wheat against *P. triticina*

**Figure 4:**
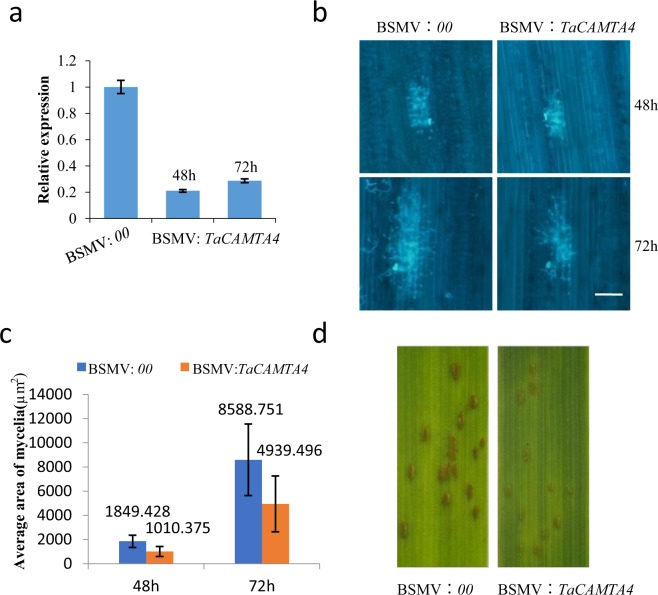
*TaCAMTA4* negatively regulate the defense response of wheat to *P*. *triticina*. (**a**) Expression analysis of *TaCAMTA4* in the third leaves inoculated with BSMV: 00 and BSMV: *TaCAMTA4* by qRT-PCR. After 12 days BSMV inoculation, the mature urediniospores of *P*. *triticina* race 165 were brushed on the newly emerged third leaves. Then the RNA extracted from the third leaves at 48 h and 72 h after *P*. *triticina* infection was subjected to qRT-PCR. (**b**) Biology microscope observations of *P*. *triticina* mycelia appearing on leaf pieces at 48 h and 72 h after inoculation. Leaf tissue was stained with 0.1% Florescent Brightener (FB) Bar = 100 μm. (**c**) The average area of mycelia was statistically analyzed between BSMV: 00(control) and BSMV: *TaCAMTA*4. Values are means (±SD) of 30 mycelia. The numbers of mycelia per visual field were counted at 48 h and 72 h after *P*. *triticina* infection. (**d**) The phenotype of uredosorus after inoculation with *P*. *triticina*. Photos were taken at 13 days after *P*. *triticina* infection.

We further hypothesized that silencing *TaCAMTA4* may enhance the defense response of wheat Lovrin 10 to rust fungus. A specific fragment of *TaCAMTA4* was cloned into the vector of BSMV to silence *TaCAMTA4* on the first leaves. BSMV:00 (BSMV empty vector) infiltrated plants were used as control in the VIGS experiment. The newly emerged third leaves were sampled at 48 h and 72 h after race 165 inoculation. To determine the effects of silencing *TaCAMTA4* gene and the resistance of wheat to *P*. *triticina*, qRT-PCR was used to detect the expression of *TaCAMTA4*. BSMV: *TaCAMTA4*-inoculated plants showed reduced *TaCAMTA4* expression levels significantly (Fig. 4a), indicating the specific suppression of the target gene in the VIGS plants.

In the meantime, the development of *P*. *triticina* hypha was observed at 48 and 72 h (Fig. 4b–d). The average area of mycelia at single infection site was much smaller and the growth rate was slower on TaCAMTA4-silenced plants compared with the control (Fig. 4b). The areas of single sorus at 8 and 13 days after infection were smaller on leaves inoculated with BSMV: *TaCAMTA4* than that on the control leaves (Fig. 4d). The chlorosis phenotype on leaves with silenced *TaCAMTA4* appeared to be more severe than the control leaves (Fig. 4d). Silencing of *TaCAMTA*4 resulted in enhanced resistance to *P*. *triticina* race 165. These results indicated that TaCAMTA4 regulate the defense response of wheat against *P*. *triticina* negatively.

## Discussion

Identifying and characterizing CaM target genes (CaMBPs), is crucial for systematically understanding the Ca^2+^-CaM signal transduction pathway in plant defense responses. A multitude of studies have confirmed that Ca^2+^-dependent CaM signal transduction pathway plays an important role in plant defense responses^17,24–26^. In the previous studies, we have preliminarily proved that Ca^2+^ was involved in the wheat defense response against *P*. *triticina*^17^. In the present study, qRT-PCR results showed CaM play an important role in the wheat defense response against *P*. *triticina*. So identification of the CaMBPs is very important for systematically study of the signaling pathway in wheat-*P*. *triticina* interaction system. Many CaMBPs have been identified, including the DNA-binding protein family, CAMTAs, by using labeled CaM to screen *A*. *thaliana* cDNA library or CaM or CML probes to detect *A*. *thaliana* protein chips^27–29^. However, functions of CAMTAs in plant defense response to pathogen were less reported. CAMTAs can bind to CaM, regulate the transcription of target genes and consequently transfer specific Ca^2+^ signals^30^. It has been reported that the six CAMTA genes in *A*. *thaliana* were induced by various abiotic stress such as cold, heat, salinity, etc.^31^. In this work, TaCAMTA4 was identified as a CaM-binding protein in the wheat-*P*. *triticina* interaction system, homologous to Arabidopsis CAMTA4 with highly conserved CAMTA domains.

The CaM-binding domain is usually on the C-terminal of CAMTAs. The CaM-binding activity of CAMTAs has already been proved in many studies. Prokaryotic expressed peptide corresponding to the CaM-binding sites of AtSR1 can bind to CaM in the present of Ca^2+^; but no binding was found^32^ in the present of EGTA. AtCAMTA1 could bind ^35^S-CaM in a Ca^2+^-dependent manner in EMSA^33^. AtSR1 is Ca^2+^-dependent CaM-binding protein and the CaM-binding site is on the C-terminal of AtSR1; moreover, all the six SRs in *A*. *thaliana* (AtSR1–6) are Ca^2+^-dependent CaM-binding proteins^31^. In this study, bioinformatic analysis indicated that the CaM-binding domain was on the C-terminal of TaCAMTA4 and highly homologous to the CaM-binding regions of many other CaM-binding proteins^8,25^. To further verify the CaM-binding sites of TaCAMTA4, we conducted phylogenetic analysis between CaM-binding domains of TaCAMTA4 and many other CaMBPs and finally chose two peptide sequences (about 30 aa) of TaCAMTA4 for CaM-binding analysis. The results of EMSA showed that the two peptide sequences could interact with TaCaM4-1 in a Ca^2+^-dependent manner. Meanwhile, the results of BiFC showed that the C-terminal of TaCAMTA4 could interact with TaCaM4-1 in *N*. *benthamiana*. Therefore, it can be concluded that TaCAMTA4 could bind TaCaM4-1 by the C-terminal CaM-binding sites in a Ca^2+^-dependent manner.

Identification of *TaCAMTA4* is very important for understanding the function of TaCAMTA4 and studying the Ca^2+^ signal transduction pathway in the defense response of wheat against *P*. *triticina*. In this work, the transcription levels of *TaCAMTA4* decreased both in incompatible and compatible combinations in the early stage of infection and decreased more obviously in incompatible combinations. In the incompatible combination, the expression level of *TaCAMTA4* reduced to the lowest at 8 h after inoculation. Our previous studies have shown that 8 h after inoculation is the time of substomatal vesicles formation. At 16 h after inoculation the primary hyphae and haustorial mother cells are formed and the haustorial mother cells contact with mesophyll cells to induce hypersensitive reaction(HR) in incompatible combination^34^. So the genes that were induced to be up-regulated or down-regulated at 8 h might involve in basic resistance. Furthermore, virus-induced gene silencing (VIGS)-based knockdown of *TaCAMTA*4 assays showed that silencing of *TaCAMTA*4 resulted in enhanced resistance to *P*. *triticina* race 165. The results indicated that *TaCAMTA4* may negatively regulate basic resistance of wheat against *P*. *triticina*. This work laid a foundation for further studies on TaCAMTA4 to better understand the interaction mechanism between wheat and *P*. *triticina*.

## Materials and Methods

### Plant materials and growth conditions

Wheat (*Triticum aesetrum* L.) variety Lovrin 10 and *P*. *triticina* race 165 made up the compatible combination, while Lovrin 10 and 260 made up the incompatible combination. *P*.*triticina* races 165 and 260 were maintained on highly susceptible wheat variety Zhengzhou 5389.

Wheat seeds were sown in 15-cm-diameter pots. Seedlings grew in the greenhouse (20–25°C, 14-h-light/10-h-dark photoperiod, 400 W/m^2^ light intensity). When seedlings grew to 7 days old with the first leaf fully expanded, the urediniospores of *P*. *triticina* were inoculated on the wheat leaves.

Tabacco seeds were sterilized in 2.5% NaClO for 15 min and washed five times with sterile water. Seeds were geminated on the MS medium in the greenhouse (20–25°C, 14-h-light/10-h-dark photoperiod) for 8 days. Then the seedlings were transferred into vermiculite and kept growing for 1 month. *N*. *benthamiana* was cultured with Hoagland nutrient solution.

### Construction of TaCaM4-1 bait vector and yeast two-hybrid screening

The full coding region of *TaCaM4-1* was amplified by PCR from cDNA of *P*. *triticina* infected wheat leaves and constructed into pGBKT7 vector in *Nde* I and *Eco*R I sites. The primers were designed based on the *CaM4-1* gene sequence of wheat variety China Spring.

The recombined vectors pGBKT7-*TaCaM4-1* was transferred into yeast AH109 by LiAc method and cDNA library screening was achieved by yeast mating. After yeast mating, the culture was inoculated on SD/-Trp-Leu-His medium and incubated at 28 °C for 7 days. Then the larger colonies were incubated another 7 days on SD/-Trp-Leu-His-Ade medium respectively followed by being transferred onto X-α-gal-based SD/-Trp-Leu-His-Ade medium for coloration detection. The colonies turned blue were regarded as possible positive colonies.

### Amplification of full-length cDNA by RACE

Total RNA was extracted from wheat leaves infected by *P*. *triticina*. Reverse transcription and RACE PCR were conducted according to the instruction of SMART^TM^ RACE cDNA Amplification Kit. 5′-RACE primers were designed according to the gene fraction sequence screened by yeast two-hybrid.

### Bioinformation analysis

The homologous genes were identified using BLAST (http://blast.ncbi.nlm.nih.gov/Blast). Protein conserved domains prediction and phylogenetic analysis were conducted in InterproScan (http://www.ebi.ac.uk/Tools/InterproScan) and ExpaSy (http://www.expasy.org). PSORT (http://psort.ims.u-tokyo.ac.jp/form/html) was for subcellular location prediction.

### CaM-binding analysis by EMSA

The synthesized peptides were dissolved in 10 mM Tris-HCl solution (pH 8.0). Peptide and prokaryotic expressed TaCaM4-1 proteins were mixed respectively in molar ratios of 0, 0.5, 1, 2, 4, and 8 in reaction buffer (10 mM Tris-HCl solution (pH 8.0) with 1 mM CaCl_2_ or 2 mM EGTA). Total reaction volume was 30 µL. After spontaneously interacting at room temperature for 1 hour, 10 µL of the reaction volume was used for 15% nondenaturing PAGE detection.

### Bimolecular fluorescence complementation (BiFC) assay

*TaCAMTA*4 and *TaCaM4-1* CDS sequences were constructed into pSPYNE (R)173 and pSPYCE (MR) respectively. Recombined plasmids were respectively transferred into *Arobacterium tumefaciens* strain GV3101. The empty vectors pSPYNE (R)173 and pSPYCE (MR), expressing split GFP domains were used as a negative control, Both pSPYNE (R)173- *TaCAMTA*4 and pSPYCE(MR)- *TaCaM4-1* were mixed, and co-transformed into *N*. *benthamiana* leaves by pressure injection method. The transformed *N*. *benthamiana* were incubated at 25 °C for 40–48 hours before observation. GFP fluorescence was recorded using a confocal laser-scanning microscope (OLYMPUS_FV1000-IX81).

### Gene expression analysis by qRT-PCR

Total RNA samples were extracted from infected leaves at 0 h, 4 h, 8 h, 12 h, 16 h, 24 h, 48 h, and 72 h after infection with *P*. *triticina*. RNA was purified and reverse transcribed to synthesis the first strand of cDNA. qRT-PCR was conducted using qRT-PCR kit (TaKaRa). Expressions of all genes were assayed for triplicated. Gene expression was calculated using the Delta-Delta cycle threshold method. qRT-PCR was also performed in mock treatment combination used as control. Consecutively expressed gene *EF-1-α* was used as a reference. Primers for qRT-PCR were designed by Primer 5 and their sequences were listed in S6 Table.

### Construction of BSMV-VIGS vector and silencing of TaCAMTA4

A 291 bp specific fragment of *TaCAMTA*4 (from 72 bp to 362 bp) was cloned to the vector of pSL038-1, to construct BSMV: *TaCAMTA4*. The primer sequences used to amplify the target gene were as follows: forward, GCGGCCGCCCAGAGAGGTTC; reverse, GCGGCCGCTCATAAGCCGGTTC. The restriction sites of *Not* I were underlined. *In vitro* transcription was performed using the mMessage mMachine™ T7 *in vitro* transcription kit (Ambion) following the manufacturer’s protocol. The tripartite BSMV RNA BSMV: α, BSMV: β, and BSMV: *TaCAMTA*4 or BSMV: 00 were inoculated on the first fully expended leave of 7–8 days old wheat lovrin 10 by rub inoculation with a gloved finger. The wheat Lovrin 10 infected with BSMV: 00 was as control. For each treatment, ten plants were inoculated.

### Virus infection and evaluation of resistance

After 12 days of BSMV inoculation,the mature urediniospores of *P*. *triticina* race 165 was brushed on the newly emerged third leaves by using a wet brush. The control wheat was treated the same simultaneously. Then the wheat were placed in a moist dark cabinet for 16 hours. Leaves were collected at 48 h and 72 h after *P*. *triticina* infection to detect the expression level of *TaCAMTA*4 by using qRT-PCR and evaluate the wheat resistance to *P*. *triticina*. Primers were designed by Primer 5 and listed in S6 Table. Fluorescent Brightener (FB) was used to dye the leaf pieces to observe the development of hypha. The area of the mycelia was measured. Photos were taken to record the phenotype of leaves and sorus at 8 days and 13 days after *P*. *triticina* infection.

## Electronic supplementary material

Dataset1, Dataset2, Dataset3, Dataset4, Dataset5, Dataset6.
