# Supplementary material for: TaCAMTA4, a Calmodulin-Interacting Protein, Involved in Defense Response of Wheat to Puccinia triticina
Source: Sci Rep. 2019 Jan 24;9:641. doi: 10.1038/s41598-018-36385-1 (PMC6345913; doi:10.1038/s41598-018-36385-1)
Supplement: Supplementary file 1 — Dataset1, Dataset2, Dataset3, Dataset4, Dataset5, Dataset6. [file 41598_2018_36385_MOESM1_ESM.pdf]

TaCAMTA4, a Calmodulin-Interacting Protein, Involved in Defense Response of Wheat to *Puccinia triticina*

Yuelin Wang[1†], Fengju Wei[1†]*, Hui Zhou[1†], Na Liu[1], Xiaonan Niu[1], Chao Yan[1], Lifeng Zhang[1], Shengfang Han[1], Chunyan Hou[1]*, Dongmei Wang[1]*

*Corresponding author: weifj98@126.com (F J. Wei); houchunyan@126.com (C Y. Hou); dongmeiwang63@126.com (D M. Wang);

S1 Figure. Yeast two-hybrid interactions between TaCAM4-1 and prey proteins.
These are the original photos in Figure 1. The same number represents the same prey protein.

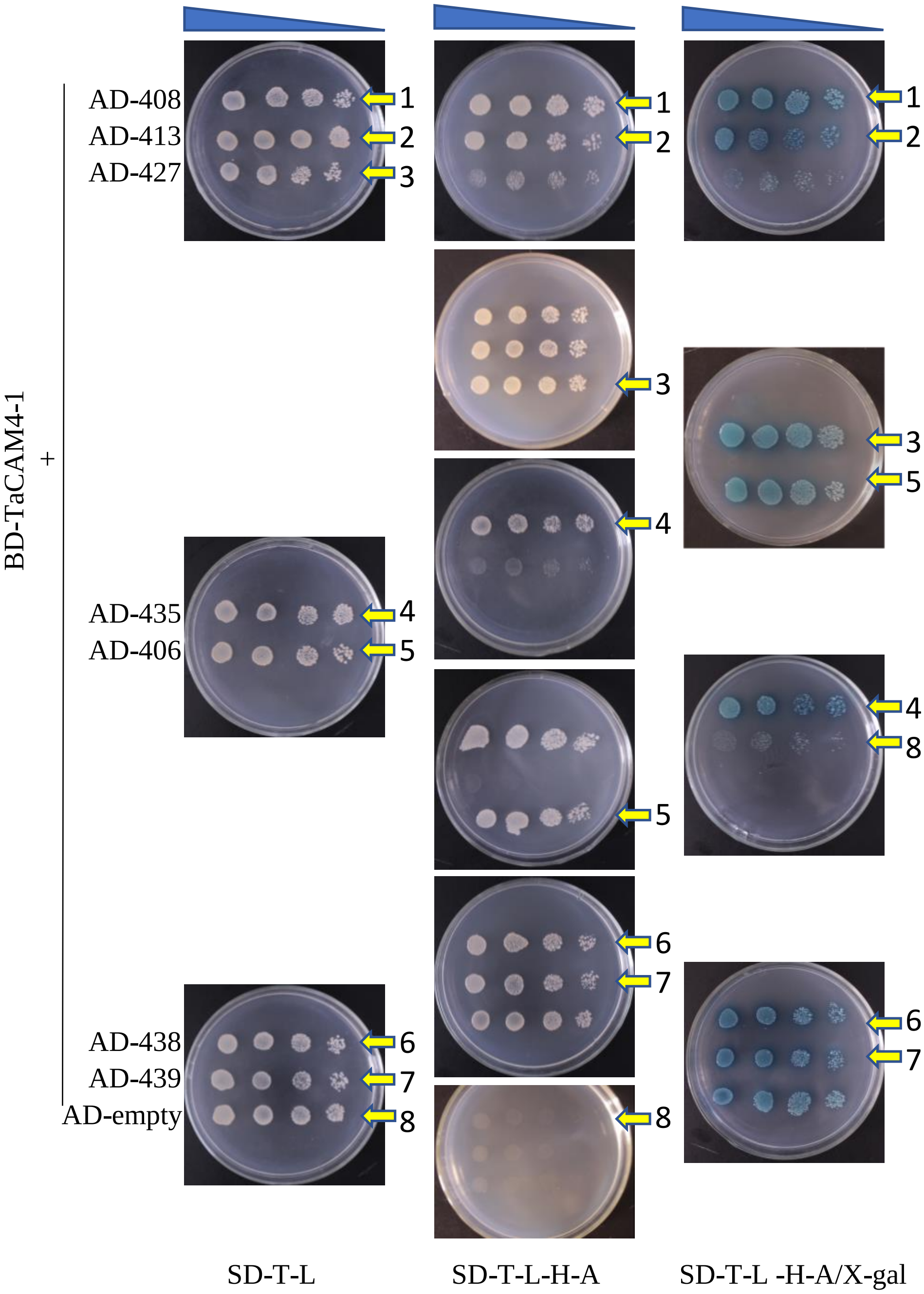

S2 Figure. TaCAMTA4 interacted with TaCaM4-1 in EMSA

These are the original photos in Figure 2 B.

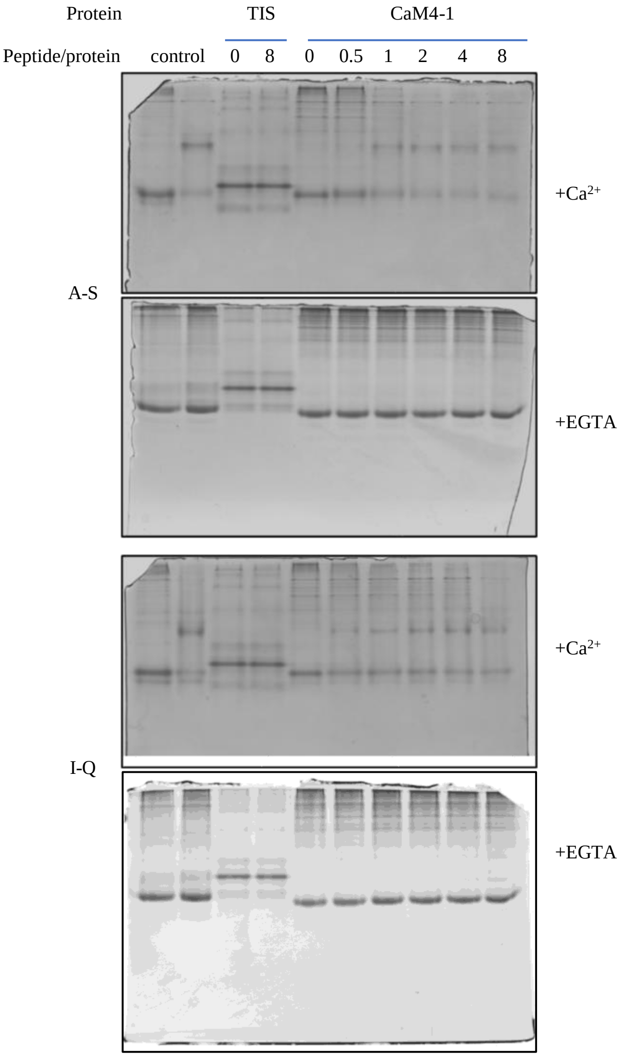

S3 Fig. TaCAMTA4 cDNA full length nucleotide sequence.

TaCAMTA4 cDNA full length was 2704 bp and the open reading frame was 2505 bp shown in gray.

```
1       ATGCAGCAGC AGCAGCGGCA AGGTCACGAC ATACTAAATC TGCAGCGGGA GGTGAAGACG
61      CGGTGGCTCA AGCCCAGAGA GGTTCTTGAC ATATTGCAGA ACTGTGAGCT GTTCGGGATC
121     CAAAACAGGA CCCCTCAGAG GCCACCGAGT GGTTCTTGGT TCCTTTTCAA CCGTAGGGTA
181     CATCGCTTCT TCCGGAATGA TGGGTATGTG TGGCAGAAAA AGAAGAATGG GAAGAGCGGC
241     AATGAAGCAC ATGAGTACCT TAAGGTTGAT AATGTAAAGG CTCTGAATTG CTACTATGCT
301     CGTGCAGAAA ATAATCCTAG ATTCATGAGG CGGATATATT GGATGCTTGA ACCGGCTTAT
361     GAGCACATCG TTCTTGTCCA CTATAGAGAC GTTCTAGAGG GCAGCATTTC AGTATCAGTG
421     CTAAACGGTT CGCCAACATC CAATCAGAAT GGCAGTGCTA GCAGAGCTGA TGCACATAGT
481     TCGCCAGGGT TGACTAGTGA GATAGTTGCG CCACTTCTCA ACTCGTGCAG CCCAGGATCC
541     GCGGAAGAAG TTAGTTCCCA GATCCCGACC ATAAACAATG AAACAAATGA TATAAGTCTA
601     TTTGATTGGC GGCGGACACT TGAAATGCAG CTGAGTCTGG AAAACAAGGA ACGTCATGAT
661     GTTAACACTG ATGAGGTTCT GCCAAATCAT GATCCTATTC CTGCGCATGG GATACAGAAT
721     GAAGAACTAG ACGCATGTAT AAACCTTGCA GATGTCTTTG ATCTGGGGTT TAGTGAAGAC
781     AACCATGCTG AAGGAAGTCA CCCTTATCCC GATCCTATCG ATGTCCTGAA ATATTCAGAA
841     ACATGGTTGG AGGATGACCA ACTTAAATCT ATTCTACATT CAGCTCCTGT GACAATTGAT
901     GAAAACCAAT GGTTCCATCT TCATGAGGTT TCTCCAGAAT GGGCATTTTG TTCTGAAAGT
961     GCTAAGGTTG TCATTGTAGG AGATTTCCCT TGCAACCTCT CCAATAGTTC ATGGGTACTA
1021    TTTGGTGATG TTAAAGTACC TGCGGTAGTT GTCCAGCAAG GTGTCATCCG TTGTTATACT
1081    CCACCATACC TTGGTGCTGG AAAGGTGAGA ATGTGCATGC TCGATGAGAA TGGGAAACCT
1141    TGCACTGAAG ATCGAGAATT TGAATTCGTT GAAAAGCCTA CCAACACAAT GATTAATGGG
1201    AACGGGAAAC CCTGCAGTGA AGCACGAGAA TTTGAATTCC AACAGAGGCC TACCAAAAGT
1261    GACAATGGGC TGTTGTTGCT GATTAACTAT GTGCAGATGC TTTTTGATAG TCATGGCTGT
1321    GAACTCTTCT CAAAGTTCAG GTTGCCACTC CCAAATGCTC GGTCTGGATT CCCAGTTAAC
1381    CCCTCAGAGA TTATAGGGAG AACATGTGAG CAGTTGGACC ATGAGAATGC AGTAAATTGC
1441    ATCATGGAAG TGATGCTTAA CAATAAGTTC CAGGACTGGC TATCATCCAA ATTTGAACAG
1501    AATAGTGAAG GGGAGTATTT GCTTCCTAGG CAATACCATG GTGTGATACA TACAATTGCT
1561    GCATTGGGAT ACGACTGGGC TTTGAAACCG CTGCTTAGTA ACGGCGTGCC TATAAACTAC
1621    CGTGATGCAA ATGGATGGAC TGCTCTGCAT TGGGCTGCAC GATTCGGAAG GGAACAAATG
1681    GTAGTGGTTC TTGTTGTTGC AGGCGCTGCT GTGGGTGCAC TTTCAGATCC AACAGCGGAA
1741    GACCCTGCTG CCAAGACACC TGCTTCGATT GCGTCTGCCT ATGGTTTCAT AGGCATCTCT
1801    GCATTCCTTT CAGAAGCACA ACTAACCAGT ACCCTTGATT CTCTGGAATC AAAAGAAAAT
1861    GGGAAGCCCG TAGATCATAA TGGTGGAGTG AGTACATCTA ATGCTGTGGA TAGAGTATCA
1921    GATAAATGTG CACATGTGGA TGGTGGAACT GATGATCAGC TTGCACTTAA GGATTCTCTA
1981    GGAGCTATCC GAAATGCTGT TCAAGCTGCC GGACGCATAC AAGCTACCTT CCGTGTGTTT
2041    TCCTTAAAAA AGAAGAAACA AAAGGCTCTT CAGAATGGAG ATAGCTCTGC TTCGCCATCT
2101    ATGCTCGAAA GAGCTACATT ATCTATCCAG AAGAACTTCA GGTGCTGGAA GAAACGTAAG
2161    GAATATCAGA AAATTCGGAA AAATGTCATC AAGATTCAGG CACGGTTCAG AGCTCACCGA
2221    GAAAGAAACA AGTACAAGGA GTTACTTCAA AGTGTTGGCA TCCTTGAGAA GATCATGCTG
2281    AGGTGGTTCC GAGAAGGTGT TGGTCTGCGA GGAATCAATA GCAGGGCGAT GCCAATCGAC
2341    CAAGACCAGA AAGAAGACAT CGTCAAGGTT TTCCGCAAGG AAAGAGTGGA AACAGCTGTC
2401    AGTGAGGCTG TTTCGAGGGT ATCGGCTATC GTCGGTTGCC CTGTCGCAAG GCTAGACTAC
2461    CGCAGGATGC TCGAAATGCA CCAACAAGCA AAGGTGAGGC CATGATCCGT GGTGTCTACT
2521    GAAGGCGTTT CAGTCAAAAA CTTGCTGAAG CAATAGCAGA TTTACGAGCG AAAAACTCAC
2581    TCCATATGTC TACTGTCTAG CACTTTGTGC ATGATATTTT GCAACTTATT TATTCTCTTG
2641    AACTTACTCA AAAAAAAAAA AAAAAAAAAA AAAAAAAACA TGTCCGCCGC CTCCGGCCTT
2701    CTAG
```

S4 Fig. Alignment of TaCAMTA4 and other homologous CAMTAs.

Comparative analysis between TaCAMTA4 and CAMTA sequences in other species. The amino acid residues that are conserved among different CAMTA proteins were colored dark blue. Gm for *Glycine max*, Vv for *Vitis vinifera*, Sl for *Solanum lycopersicum*, Bd for *Brachypodium distachyon*, Os for *Oryza sativa* Japonica Group.

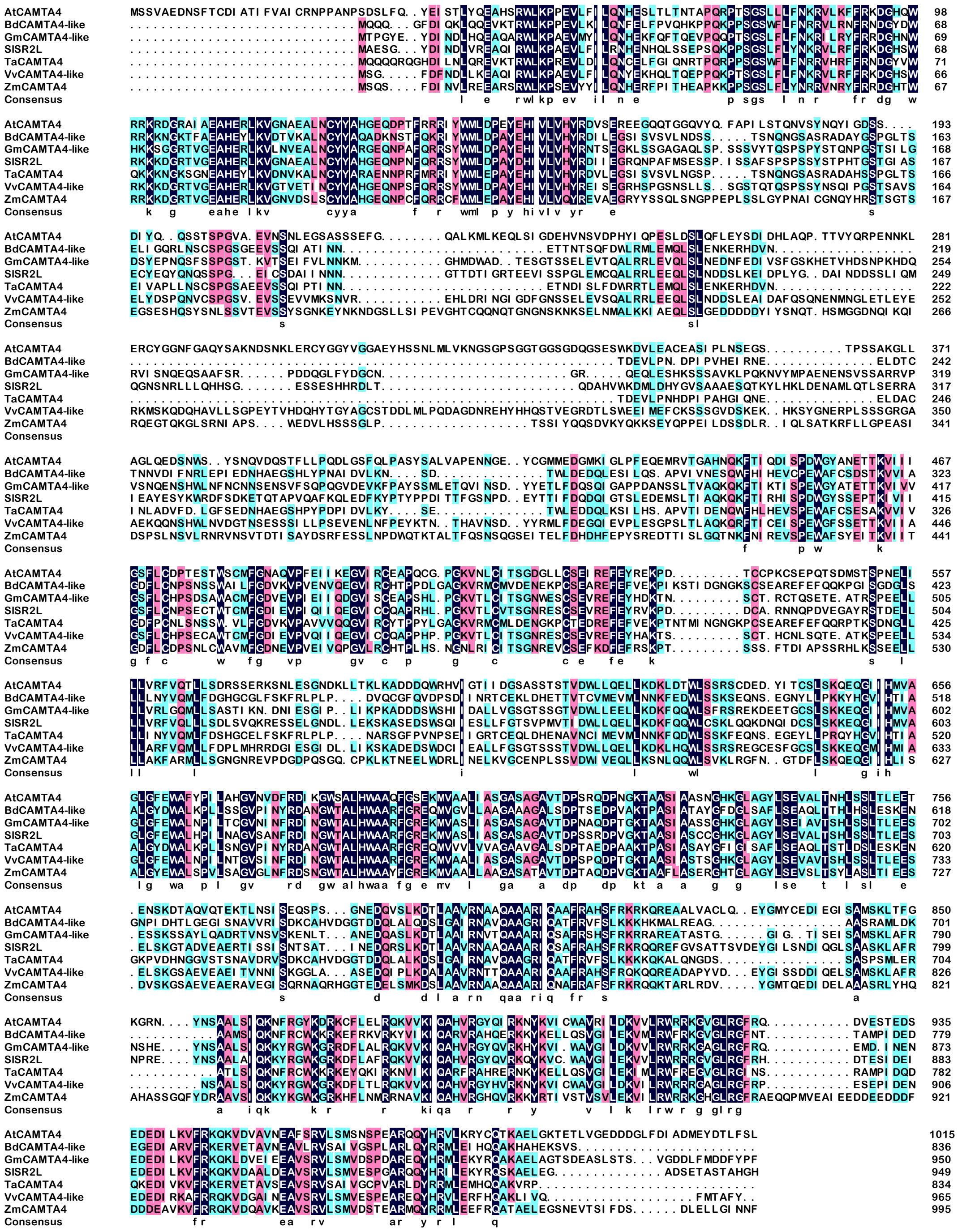

S5 Table. Blastn results of *TaCaM4-1* screened genes.

Gene Code	Description	Code	Number
AK332814.1	*Triticum aestivum* cDNA, clone: SET1_O14, cultivar: Chinese Spring	406	1
AK374070.1	*Hordeum vulgare* subsp. Vulgare mRNA for predicted protein, complete CDS, clone: NIASHv16	408	6
AK334410.1	*Triticum aestivum* cDNA, clone: WT009_J02, cultivar: Chinese Spring	413	1
AK331952.1	*Triticum aestivum* cDNA, clone: WT002_M11, cultivar: Chinese Spring	427	1
AK252052.1	*Hordeum vulgare* subsp. vulgare cDNA clone: FLbaf143h10	435	2
	No significant similarity found	438	3
AK335614.1	*Triticum aestivum* cDNA, clone: WT013_F15, cultivar: Chinese Spring	439	3

S6 Table. Principal primers used in this study.

Primers name	Sequence
TaCAMTA4 （For 5’RACE）	F: CACCTTCTCGGAACCACCTCAGC<br>R: CGAGCATAGATGGCGAAGCAGAG
TaCAMTA4 （qRT-PCR）	F: CTCTGCTTCGCCATCTATG<br>R: CCGTGCCTGAATCTTGAT
TaCAMTA4 （DNA-binding）	F: CGGAATTCCGGCAAGGTCACGACATAC<br>R: CCCAAGCTTTGTTGGCGAACCGTTTAG
EF1a （RT-qPCR）	F: ATGCTGGCATAGTGAAGATG<br>R: AACAGTAGCAAAACTTGCAC
TaCAMTA4（qRT-PCR，VIGS）	F: ATCCGTGGTGTCTACTGAAG<br>R: TCATGCACAAAGTGCTAGAC
